# From *In Vitro* to Perioperative Vascular Tissue Engineering: Shortening Production Time by Traceable Textile-Reinforcement

**DOI:** 10.1007/s13770-022-00482-0

**Published:** 2022-10-06

**Authors:** Saurav Ranjan Mohapatra, Elena Rama, Christoph Melcher, Tobias Call, Miriam Aischa Al Enezy-Ulbrich, Andrij Pich, Christian Apel, Fabian Kiessling, Stefan Jockenhoevel

**Affiliations:** 1 Department of Biohybrid and Medical Textiles (BioTex), Center for Biohybrid Medical Systems (CBMS), Institute for Applied Medical Engineering, RWTH Aachen University, Forckenbeckstr. 55, 52074 Aachen, Germany; 2 Institute for Experimental Molecular Imaging, RWTH Aachen University, Forckenbeckstr. 55, 52074 Aachen, Germany; 3 Institute for Textile Technology, RWTH Aachen University, Otto-Blumenthal-Str. 1, 52074 Aachen, Germany; 4 DWI-Leibniz Institute for Interactive Materials, RWTH Aachen University, Forckenbeckstr. 50, 52074 Aachen, Germany

**Keywords:** Tissue-engineered vascular grafts, Electrospun scaffolds, Non-invasive monitoring

## Abstract

**Background:**

The production of tissue-engineered vascular graft (TEVG) usually involves a prolonged bioreactor cultivation period of up to several weeks to achieve maturation of extracellular matrix and sufficient mechanical strength. Therefore, we aimed to substantially shorten this conditioning time by combining a TEVG textile scaffold with a recently developed copolymer reinforced fibrin gel as a cell carrier. We further implemented our grafts with magnetic resonance imaging (MRI) contrast agents to allow the *in-vitro* monitoring of the TEVG’s remodeling process.

**Methods:**

Biodegradable polylactic-*co*-glycolic acid (PLGA) was electrospun onto a non-degradable polyvinylidene fluoride scaffold and molded along with copolymer-reinforced fibrin hydrogel and human arterial cells. Mechanical tests on the TEVGs were performed both instantly after molding and 4 days of bioreactor conditioning. The non-invasive *in vitro* monitoring of the PLGA degradation and the novel imaging of fluorinated thermoplastic polyurethane (^19^F-TPU) were performed using 7T MRI.

**Results:**

After 4 days of close loop bioreactor conditioning, 617 ± 85 mmHg of burst pressure was achieved, and advanced maturation of extracellular matrix (ECM) was observed by immunohistology, especially in regards to collagen and smooth muscle actin. The suture retention strength (2.24 ± 0.3 N) and axial tensile strength (2.45 ± 0.58 MPa) of the TEVGs achieved higher values than the native arteries used as control. The contrast agents labeling of the TEVGs allowed the monitorability of the PLGA degradation and enabled the visibility of the non-degradable textile component.

**Conclusion:**

Here, we present a concept for a novel textile-reinforced TEVG, which is successfully produced in 4 days of bioreactor conditioning, characterized by increased ECM maturation and sufficient mechanical strength. Additionally, the combination of our approach with non-invasive imaging provides further insights into TEVG’s clinical application.

**Supplementary Information:**

The online version contains supplementary material available at 10.1007/s13770-022-00482-0.

## Introduction

Cardiovascular diseases often demand instant care but producing a suitable solution quickly is highly challenging. Replacement of diseased vessels with synthetic grafts or autologous grafts are two main therapy options for these life-threatening conditions. Several polymeric matrices have been developed and studied for use as artificial grafts in vascular applications, including polyurethane (PU), expanded PTFE (ePTFE), and poly(ε-caprolactone) (PCL) [1–3]. With the aid of balloons and catheters, vascular bypasses using autografts, artificial grafts, or vascular stents have been widely studied [4–6]. Though clinically established synthetic grafts are currently in use, the indications are limited to higher vascular diameters, and susceptibility to infections can lead to morbidity and mortality [7–9]. In the past few years, tissue engineering has made tremendous advancements, and both *in vitro* and *in vivo* studies have been successfully realized [10]. In combination with polymer scaffolds and autologous cells, tissue engineering has the potential to create body-own replacement structures. Tissue-engineered constructs hold the ability for self-repairing and remodeling [11].

Besides the advancement in vascular tissue engineering, the long *in vitro* culture time remains a big challenge with regard to a successful translation into clinics [12]. It has been reported that TEVGs require two to three months of culture to give a desired implantable condition [13–16]. To serve an acute and subacute case of cardiovascular dysfunction this time is impractical. The conditioning time is given not only to achieve higher cell density but also to bring higher burst strength. An immature extracellular matrix can lead to the development of life-threatening aneurysms in short to medium term [17].

Previous studies have already shown that conditioning the grafts *in vitro* with an oscillating pressure increases the burst pressure over time. However, in those studies, the graft had to be kept in a bioreactor for several weeks to demonstrate suitable mechanical properties for implantation. Gui et al. and Stekelenburg et al. reported a burst pressure of 913.3 ± 150.1 mmHg after 30 days of culture under pulsatile stretching and 903 ± 123 mmHg after 28 days of *in vitro* culture, respectively [18, 19].

Two important factors have to be considered to make a successful TEVG. The first factor that possesses the mechanical stability of the TEVG must imitate the robustness of a native vessel. Second, adhesive and confluent endothelial cells in the lumen area to avoid thrombosis after implantation [20].

**Fig. 1:**
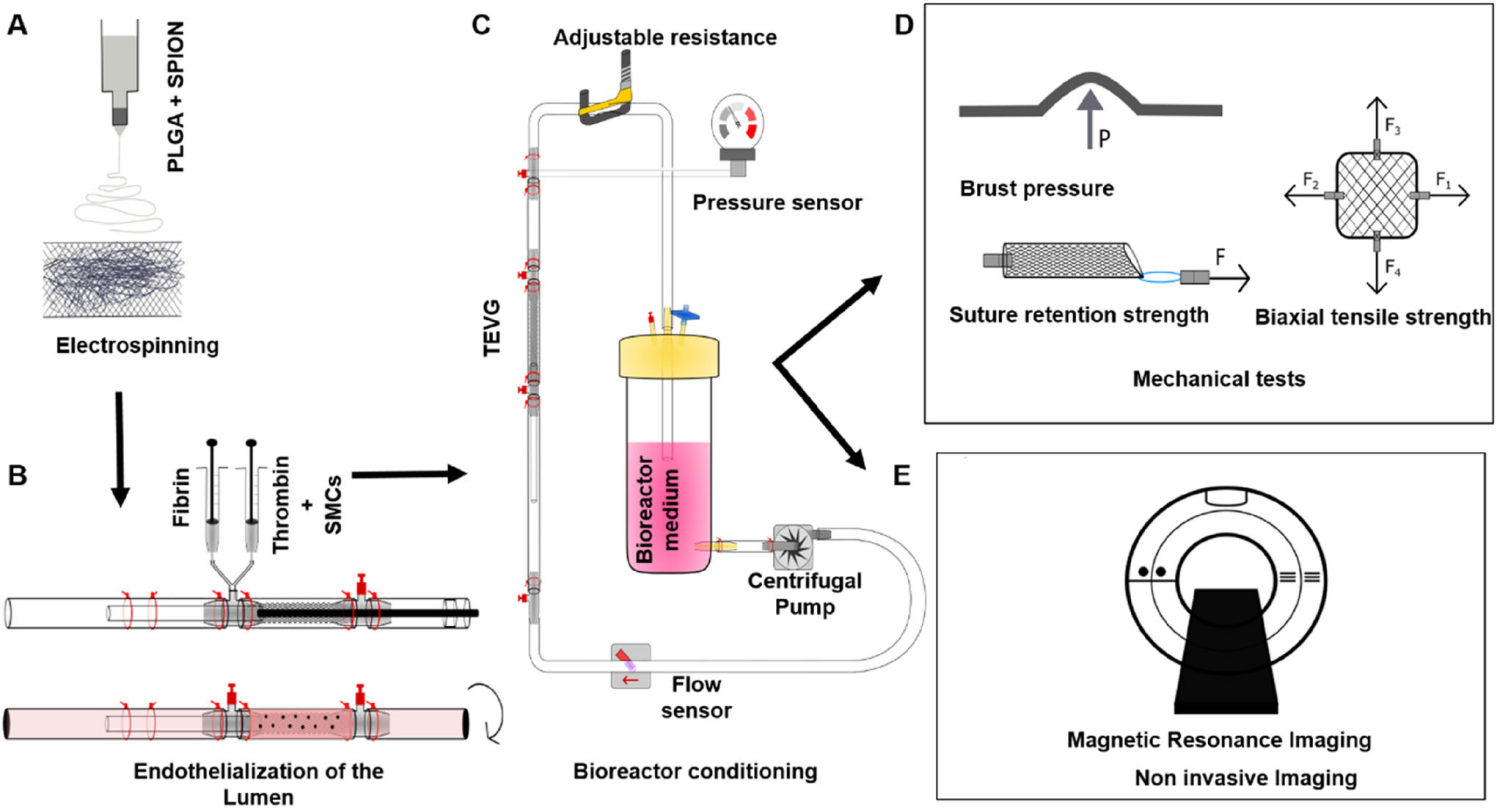
Schematic representation of the study design. **A** Electrospinning of the PLGA layer on the textile mesh, **B** Molding technique of the TEVG, **C** The closed loop bioreactor, **D** Mechanical tests, **E** Non-invasive Imaging methods

We have invented the concept of a textile-reinforced biohybrid vascular graft and, till now, have successfully applied this concept *in vitro* and *in vivo* [21–24]. However, the current challenge is to reduce the bioreactor conditioning time significantly to foster translation into the clinic. We hypothesize that this could be achieved by either adding an improved textile reinforcement and/or modifying the hydrogel cell carrier. In the present study (Fig. 1), we are employing a non-degradable textile poly(vinylidene fluoride) (PVDF) mesh to give long-term structural support to avoid aneurysm formation. The PVDF textile mesh is coated with an electrospun poly(lactic-*co*-glycolic-acid) (PLGA) layer to give temporary higher burst strength. The nonwoven PLGA meshes fill up the gaps between textile filaments and introduce microporosity to the graft. The electrospun layer of PLGA has proven to be biocompatible and biodegradable. Apart from that, it is cost-efficient and easily producible [25–27].

Furthermore, we modified the fibrin matrix gel by adding reactive amphiphilic copolymers, which are based on *N*-vinylpyrrolidone (VP). *N*-vinyllactam-based polymers have been proven biocompatible and possess specific hydration properties [28]. Pich et al. have successfully improved the mechanical properties of fibrin and thrombin-based hydrogels by adding the poly(*N*-vinylpyrrolidone-*co*-glycidyl methacrylate) (VP-*co*-GMA) copolymer [29]. We hypothesize that the integration of this copolymer will enhance the initial mechanical strength of the vascular grafts.

Magnetic resonance imaging (MRI) is a non-destructive approach and can be used in both *in vitro* and *in vivo* monitoring. Cardiovascular functions can be broadly investigated by the MRI device because of its optimum contrast of soft tissues [30]. As discussed in a previous study, the incorporation of MRI contrast agents could be exploited as an additional technique to improve vascular grafts’ visibility via non-destructive imaging [31]. Non-destructive monitoring of implants provides not only real-time diagnostics but also ensures patient safety. Mertens et al. have successfully shown the enhanced visibility of collagen scaffolds using ultrasmall superparamagnetic iron oxide nanoparticles via 3 T MRI [32]. In addition, Rama et al. have recently shown that after superparamagnetic iron oxide nanoparticles (SPION)-labeling of their vascular grafts’ textile scaffold, the longitudinal monitoring of the degradation and remodeling of vascular prostheses is accessible via multimodal imaging [33]. Furthermore, as a valuable alternative to ^1^H MRI, Lammers et al. already showed the potential that could result from the employment of highly fluorinated thermoplastic polyurethane (^19^F-TPU) as a novel material for tissue engineering applications [34]. Indeed, ^19^F-MRI techniques might provide useful insight, especially for quantitative applications, thanks to the lack of ^19^F signals in human or animal bodies.

In this study, we present tissue-engineered vascular grafts, which would be ready to be transplanted after only 4 days of bioreactor conditioning with well-suited mechanical rigidity to allow implantation. We compared three types of TEVG: (i) *Non-coated TEVG:* contains a not-coated PVDF textile mesh and fibrin matrix hydrogel along with cells (ii) *Coated TEVG*: contains an electrospun PLGA coated PVDF textile mesh and fibrin matrix hydrogel along with cells and (iii) *Copolymer TEVG*: contains VP-*co*-GMA copolymer along with the coated PVDF textile mesh, fibrin, and cells. The three variants of TEVGs were mechanically tested and histologically analyzed instantly after molding (day 0) and after four days of bioreactor conditioning (day 4). In addition to that, we demonstrate the employment of novel imaging approaches allowing the traceability and monitorability of vascular grafts. Therefore, we aimed to employ non-invasive and non-destructive ^1^H/^19^F MRI to investigate and monitor our TEVGs’ integrity and remodeling. To this end, we also introduced SPION and ^19^F-TPU into the textile scaffold of our vascular grafts.

## Materials and methods

### Production of textile mesh

As already described in our previous publication [33], a double-bar Raschel warp-knitting machine (Karl Meyer Holding GmbH & Co. KG, Obertshausen, Germany) was used to produce three different kinds of tubular meshes structures from polyvinylidene fluoride (PVDF) (Lenzing Plastics GmbH & Co. KG, Lenzing, Austria) multifilament fibers (150 dtex, 48 filaments). To alter the inner diameters of the tubular mesh structures, the number of used fibers was varied (4, 6, and 10 fibers). The fabrication parameters and effective arc per centimeter (EAC) value of the warp-knitting process are shown in Table 1.

**Table 1:** Warp-knitting process parameters for the tubular PVDF mesh structures

Region of the mesh	Chain notation of warp-knitting	EAC value
Brim fibers	0-2-2-2-2-2-0-2-0-2-2-2-2-2-0/2// 0-0-2-0-2-0-0-0-0-0-2-0-2-0-0-0//	6
Back part	2/2-2-0-2-2-2-4-2-2-2-0-2-2-2/4//	6
Front part	0-2-2-2-2-2-0-2-0-2-2-2-2-2-0/2//	6

#### ^19^F-TPU fiber fabrication

^19^F-TPU (Lubrizol, Wickliffe, Ohio, United States) multifilaments were produced via melt spinning (Fourné Polymertechnik GmbH, Alfter, Germany). ^19^F-TPU pellets were dried at 80 °C for 24 h prior to spinning using a vacuum oven (Thermofischer Scientific, Waltham, MA, USA). For extrusion of the fibers, extrusion temperatures (T_E_) of T_E_ = 185–220 °C were used. The multifilament spinnerets provided 10 circular shaped capillaries. The spinning line setup consisted of a blow chamber, a mono godget, two godget pairs, a heat channel as well as a winder (SAHM 260XE, Georg Sahm GmbH & Co. KG, Eschwege, Germany) for fiber collection. The heating channel was placed between the two godget pairs and heated to a temperature of 65 °C. VP 3 GA 661/19 (20 vol.-% in deinoized water) was used as a spin finish for multifilament fabrication. Multifilaments could be produced at spinning velocities (v_S_) of v_S_ = 130–144 m/min.

### Electrospinning of SPION-labelled PLGA fibers

Warp-knitted meshes were coated with SPION-labelled PLGA (Purasorb PLG 8523, Corbion Purac Gorinchem, Netherlands) electrospun fibers for 10 min. Therefore, a coaxial spinning head (Bioinicia SL, Paterna, Spain) with a 1400 µm shell capillary and a 580 µm core capillary was used. For each spinning trial, the two used spinning dopes (6 wt% PLGA (shell) and 6 wt% PLGA labeled with 0.2 wt% SPIONs (core), dissolved in methanol (MeOH, neoLab Migge Laborbedarf – Vertriebs GmbH, Heidelberg, Germany) and chloroform (CHCl3, Carl Roth GmbH & Co. KG, Karlsruhe, Germany) were prepared in a total volume of 10 ml and stirred for 24 h at room temperature until the PLGA was completely dissolved. The SPION solution was added to the core solution directly before execution of the electrospinning process and stirred until reaching a homogenous solution. For coating, the PVDF meshes, 30 cm of the structures were applied on a cylindrical collector. The tip to collector distance was set to 20 cm, and the printing head was driven continuously along the PVDF mesh at a speed of 30 mm/s. The flow rates of the core and shell solutions were set to 0.5 ml/h and 1 ml/h during the spinning process. SPION labeled PLGA fibers were spun at a voltage of + 22 kV (emitter) and − 20 kV (collector). All spinning trials were performed at 25 °C and 30% humidity.

### Scanning electron microscopy

For microfiber and graft observation, a scanning electron microscope (SEM – 606 LV (JEOL, Tokyo, Japan) was used. The operating voltage was set to 15 kV; images were taken at 100-, 200-, 500- and 1000-fold magnification. To avoid charging effects and ensure electrical conductivity, the specimen was coated with a thin layer of Au/Pt before measuring the SEM images.

### Cell isolation

The human umbilical cord was collected in a transport buffer solution beaker and kept at 4 °C for 4 h. The umbilical cord was cleaned properly with PBS, and the remaining blood clots were removed. To harvest the smooth muscle cells, the arteries were isolated from the cord and minced into small ring-shaped pieces with a scalpel. The small pieces were distributed horizontally inside a T75 cell culture flask and supplied with a fresh DMEM medium (Thermofischer Scientific).

For the endothelial cells, the lumen of the HU artery was soaked with collagenase (Thermofischer Scientific) for 30 min at 37 °C for incubation. After the collagenase removal, the cells were cultured in the flask and supplied with an endothelial basal medium (Promocell, Heidelberg, Germany). The umbilical cord was collected from the Gynecology Department at the University Hospital Aachen in accordance with the human subjects’ approval of the ethics committee (vote of the local ethics committee: ‘EK 2067).

### Tissue-engineered vascular graft

To prepare the TEVG, a molding system was created as described previously [21]. The molding system consists of a molding cavity and a cylindrical core. The molding cavity is made of a 6.4 mm inner diameter silicon tube (Carl Roth), and a 3 mm solid steel rod was employed as the core. Both ends of the mold were connected to T-connectors with Luer-lock (Fleima Plastic, Wald-Michelbach, Germany), which were used as sprue and riser. Each sample of the TEVGs are 4 cm long, the inner diameter is 3 mm, and the outer diameter is 6.4 mm. The hydrogel inserted into the mold (375 µl/cm) was a combination of fibrinogen (10 mg/ml) (Thermo Fisher), thrombin (Sigma-Aldrich, Darmstadt, Germany), TBS buffer (Thermo Fisher), CaCl_2_ (Sigma-Aldrich) and arterial smooth muscle cells (10 × 10^6^/ml). The polymerization following the molding was confirmed, and the metal core was removed afterward. The lumen was filled with arterial endothelial cells at a concentration of 3 × 10^6^/ml. Then the TEVGs were constantly rotated at 1 rpm for 6 h, using a modified peristaltic pump (Ismatec, Wertheim, Germany) to equally distribute the endothelial cells on top of the surface of the lumen. The copolymer TEVGs were prepared separately, and along with the above-described fibrin matrix gel, the VP-*co*-GMA copolymer solution (40 µl per TEVG) was added. The copolymers were produced via RAFT polymerization as described previously by Peng et al. and Pich et al. [29, 35].

A custom-made close-loop bioreactor system was designed to circulate the medium continuously. A centrifugal pump (Rs Pro, Plymouth, UK) was attached to transport the media. A pressure sensor (Codan, Lensahn, Germany) and a flow computer (Em-tec GmbH, Finning, Germany) were attached to the bioreactor system. The pressure was maintained from 80 to 120 mmHg, and the flow rate was maintained from 50 to 200 ml/min. Additionally, a clamp (Bürkle GmbH, Bad Bellingen, Germany) was installed to adjust the pressure within the system.

### Synthesis of VP-*co*-GMA-copolymers via RAFT polymerization

First, the RAFT agent Rhodixan A1 was synthesized as described in the literature [36, 37]. Methyl 2-bromopropionate (Sigma-Aldrich; 40 g, 234 mmol, 1 equiv.) was dissolved in ethanol (300 ml). Potassium ethyl xanthogenate (Sigma-Aldrich; 43.1 g, 268 mmol, 1.1 equiv.) was added stepwise under ice-cooling to the solution within 45 min. Then, the solution was stirred at room temperature for 3 h. The reaction mixture was then filtered and concentrated at 40 °C at a pressure of 175 millibars. Dichloromethane (Sigma-Aldrich; 600 ml) was added, then the organic phase was extracted with demineralized water (4 × 100 ml) before it was dried over magnesium sulfate (Sigma-Aldrich) for 12 h. The solvent was removed under reduced pressure. The RAFT agent was dried under a high vacuum for 8 h.

The copolymers were synthesized via RAFT polymerization as described previously by Peng et al. [29, 35]. The synthesis was performed under an inert atmosphere. For a copolymer with a target molecular weight of approximately 15 000 g/mol and a glycidyl methacrylate (GMA) content of approximately 10 mol%, first, *N*-vinylpyrrolidone (VP, TCI chemicals) and GMA (TCI chemicals) were purified by vacuum distillation to remove the inhibitor. VP (4 g, 36 mmol, 1 equiv.) and the RAFT agent (0.063 g, 0.30 mmol) were dissolved in anisole (Sigma-Aldrich; 6 ml) and degassed with five freeze–pump–thaw-cycles. Then, the reaction temperature was set to 60 °C. A GMA solution (0.51 g, 3.6 mmol, 0.1 equiv.) in anisole (2 ml) and an initiator solution (AIBN, 0.0099 g, 0.060 mmol, 0.002 equiv.) in anisole (0.4 ml) were prepared and degassed with a nitrogen flow under ice-cooling for 30 min. To start the polymerization, the initiator solution was added to the reaction mixture. The GMA solution was added continuously by a syringe pump with a rate of 0.15 ml h^−1^ to the reaction flask. The mixture was cooled down with liquid nitrogen after 24 h to stop the polymerization. The copolymer was precipitated in diethyl ether (400 ml) under ice cooling and dried under vacuum at 40 °C for 36 h.

### Mechanical properties

To measure the burst strength, the TEVGs were cut and opened into rectangular shaped 1cm^2^ samples and kept inside a custom-made liquid-tight burst chamber. A pressure sensor (Jumu, Munich, Germany), power supply unit and data acquisition I/O device (National Instruments, Austin, TX, USA) were assembled to detect the pressure curve. The sample was kept in between two O-rings in the burst chamber to confirm the proper sealing, and PBS buffer was pumped into the chamber at the rate of 7.5 ml/min via a peristaltic pump (Ismatec) until the sample burst. The pressure courses were recorded via Labview software (National Instruments).

#### Biaxial strength

To measure the biaxial strength, the TEVGs were cut and sectioned into 1 × 1 cm samples. The samples were clamped from both longitudinal and transversal directions and were pulled from all four sides at a rate of 10 mm/min until the failure point (Zwick Roell, Ulm, Germany). The data were recorded via the test expert software (Zwick Roell). The stress values were calculated by dividing the force by area, and the strain values were calculated by dividing the elongated length by the original length. The young's modulus (Y) values were calculated from the slope of the stress–strain curve. The statistical analysis was performed using one way ANOVA (mean ± SD) with Tukey post hoc corrections.

#### Suture retention strength

The TEVGs samples were cut diagonally on one side where the suture loop was placed. A size 7.0 suture (Ethicon, USA) was placed 2 mm from the edge according to the DIN EN ISO 7198-A.5.7.4.2.. One side of the TEVG was clamped, and the suture was connected to the uniaxial device (Zwick Roell) and pulled at a rate of 50 mm/min.

#### Statistical analysis for mechanical tests

For the above-mentioned mechanical tests, all samples (sample size = 6) were analyzed by applying a one-way analysis of variance(ANOVA) with Tukey post hoc correction using Origin Pro software (Northampton, MA, USA).

### Magnetic resonance imaging

A PVDF warp-knitted structure was coated with 0.2% (w/w) SPION-labeled PLGA and then combined with a different number of ^19^F-TPU fibers (Lubrizol, Wickliffe, OH, USA). The textile structures were embedded in 10% gelatin (w/v) and then imaged in a Bruker BioSpec 70/20 USR 7T MRI (Bruker BioSpin GmbH, Rheinstetten, Germany) equipped with a dual-tuned 1H/^19^F transmit/receive volume coil. After obtaining the ^19^F-NMR spectra of the samples, which showed two distinct peaks at − 84.92 and − 78.18 ppm, the offset frequency was determined and set at − 24.000 Hz.

An ultrashort echo time (UTE) sequence [repetition time: 30 ms; echo time: 0.296 ms; flip angle: 15.0°; averages: 100; matrix size: 32 × 32; field of view: (40 × 40) mm^2^; slice thickness: 5 mm] was used to image the ^19^F-TPU fibers. In addition, an initial trajectory measurement based on ^1^H was performed to reduce distortions caused by the not-so-abundant ^19^F signal. Qualitative T2-weighted (T2W) images were acquired using a fast spin echo sequence [echo time: 80 ms; repetition time: 4000 ms]. The superimposition and the 3D-rendering of the ^1^H and ^19^F MRI images were obtained using the Imalytics Preclinical Software (Gremse-IT GmbH, Aachen, Germany).

### Immunohistochemistry

Based on the protocol described by Koch et al., Carnoy’s solution was used to fix the samples [38]. Subsequently, they were washed in ethanol, dehydrated using a dehydration device (Leica TP 1020, Wetzlar, Germany), and embedded in paraffin until further use. Paraffin sections were cut at 5 µm thickness using a microtome (Microm HM 430, Thermofisher Scientific). Before staining, the samples were deparaffinized by serial dilution of xylol and ethanol. Sequenza Staining racks (Thermofisher Scientific) were used to hold in place the samples. 0.1% Triton X-100 aqueous solution containing 5% of normal goat serum (NGS) (Agilent Dako, Santa Clara, CA, USA) was used to block and permeabilize the tissues. Afterward, the primary antibodies were incubated at 37 °C for 1 h, and the samples were washed 3 times with PBS for 5 min and then incubated with the respective secondary antibodies for 1 h. After washing the samples 3 times, they were incubated with DAPI (1:500) (Thermofisher Scientific) and mounted with a fluorescent mounting medium (Agilent Dako). The primary antibodies employed for the histological analyses are monoclonal, anti-human alpha-smooth muscle actin (α-SMA) (Sigma-Aldrich); monoclonal, anti-human CD31 (Sigma-Aldrich); polyclonal, anti-human type I collagen (Acris Antibodies GmbH, Herford, Germany); polyclonal, anti-human type IV collagen (Acris Antibodies GmbH); polyclonal, anti-human elastin (Acris Antibodies GmbH). The secondary antibodies are anti-mouse Alexa Fluor 488, anti-mouse Alexa Fluor 594, and anti-rabbit Alexa Fluor 594 (Thermofisher Scientific). An Axio Imager M2 fluorescence microscope equipped with an AxioCam MRm Rev.3 camera (Carl-Zeiss, Oberkochen, Germany) with a magnification of 20 × was used to acquire the most representative images of each stained section.

#### Quantitative analysis of immunohistology

All the images used for the quantification of collagen-1, collagen-4, α-SMA, and CD31 were acquired using a fixed exposure time for each channel. Subsequently, the images were post-processed with the AxioVision software (Carl-Zeiss) and quantified via ImageJ (NIH and LOCI, University of Wisconsin) to ensure the same windowing and thresholding.

#### Statistical analysis

All quantified data of histological samples (sample size = 3) were analyzed by applying one-way analysis of variance(ANOVA) with Tukey post hoc correction using Origin Pro software (Massachusetts, USA).

## Results

### Electrospinning and molding of the TEVGs

**Fig. 2:**
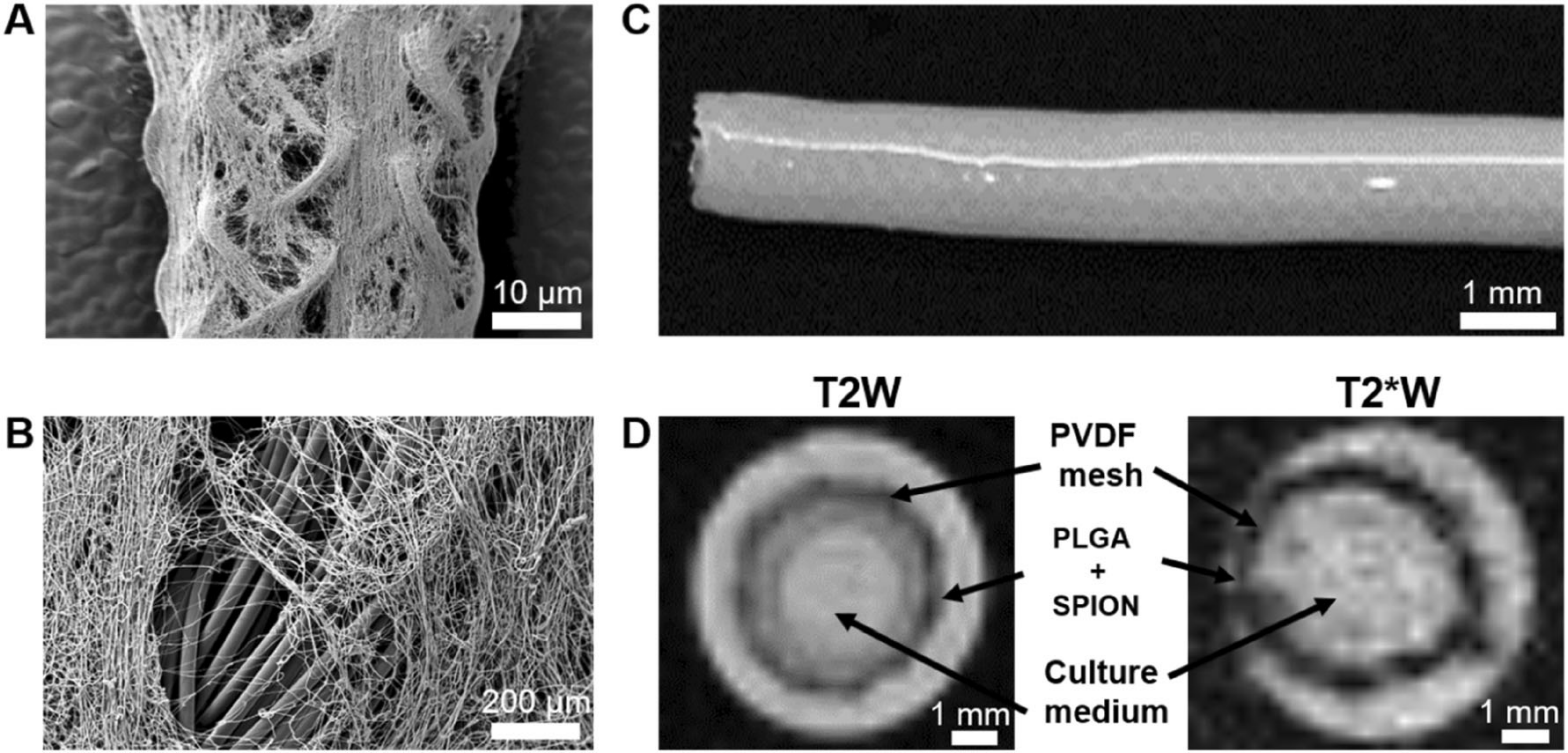
Electrospun PLGA coated TEVG. **A**, **B** SEM images of the PLGA and SPION coating on the textile mesh. **C** TEVG after molding. **D** MR images showing the lumen area and the coating upon the textile mesh

The observation of the orientation and deposition of the fibers was performed by scanning electron microscopy (SEM). The SEM images (Fig. 2A, B) confirm the fiber spreading and the porosity caused by the electrospun coating. There was no evidence of fiber agglomeration or ‘beads on string’ structure all over the textile scaffold. After molding, the MR imaging confirms a concentric layer of fibrin gel and PLGA + SPION coating on the PVDF mesh (Fig. 2C, D.

### Mechanical strength of the TEVGs

**Fig. 3:**
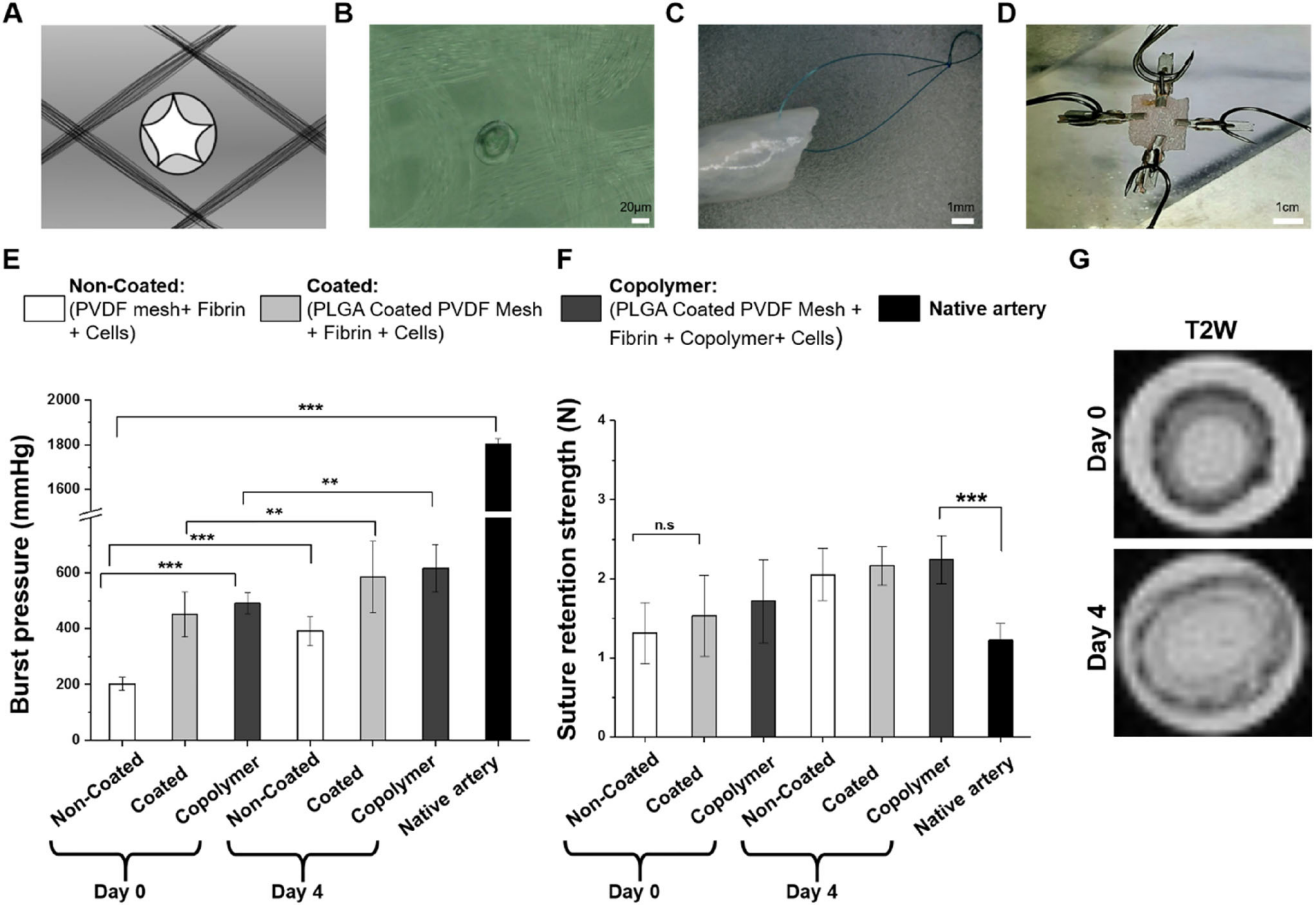
Mechanical properties of the TEVG. **A** Schematic and representation of hole-like rupture on the TEVG after the burst test between the fibers through the hydrogel. **B** Microscopic image of the ruptured hole between the fibers. **C** The suture loop on a TEVG sample. **D** Biaxial tensile test of a rectangular cut sample. **E** Burst pressure measurement of the TEVGs. **F** Suture retention strength of TEVGs. **G** Qualitative T2W images of SPION-labeled PLGA coating after 0 and 4 days of bioreactor conditioning. The hypointense signal generated by the SPION decreased over time, correlating with the PLGA coating degradation. Statistical analysis was performed using one way ANOVA (mean ± SD) with Tukey post hoc corrections (n.s *p* > 0.05, *p ≤ 0.05, **p ≤ 0.01, ***p ≤ 0.001)

The e-spun coated TEVGs and copolymer TEVGs show significantly higher burst strength than the non-coated TEVGs (n = 6) (Fig. 3E). The instantly molded TEVGs (day 0) show lower burst strength whereas, after four days of bioreactor conditioning (day 4), the burst strength shows a notable increase. The highest burst strength achieved was in the copolymer TEVGs at day 4 (617 ± 85 mmHg). The burst strength value of the native artery was found to be (1803 ± 26 mmHg).

All sets of TEVGs (n = 6) had higher suture retention strength than the native artery (Fig. 3F). The bioreactor conditioning (day 4 TEVGs) enhanced the suture retention strength, but the electrospun coating does not play an important role in amplifying the suture retention strength as the increase is non-significant. However, the addition of copolymer on the TEVGs increased the burst strength significantly (Fig. 3E). The signal of the SPION-labeled PLGA coating was observed and qualitatively studied via 7T MRI. After 4 days of bioreactor conditioning, the samples (n = 3) showed decreased signal intensity in comparison to day 0 (Fig. 3G).

**Fig. 4:**
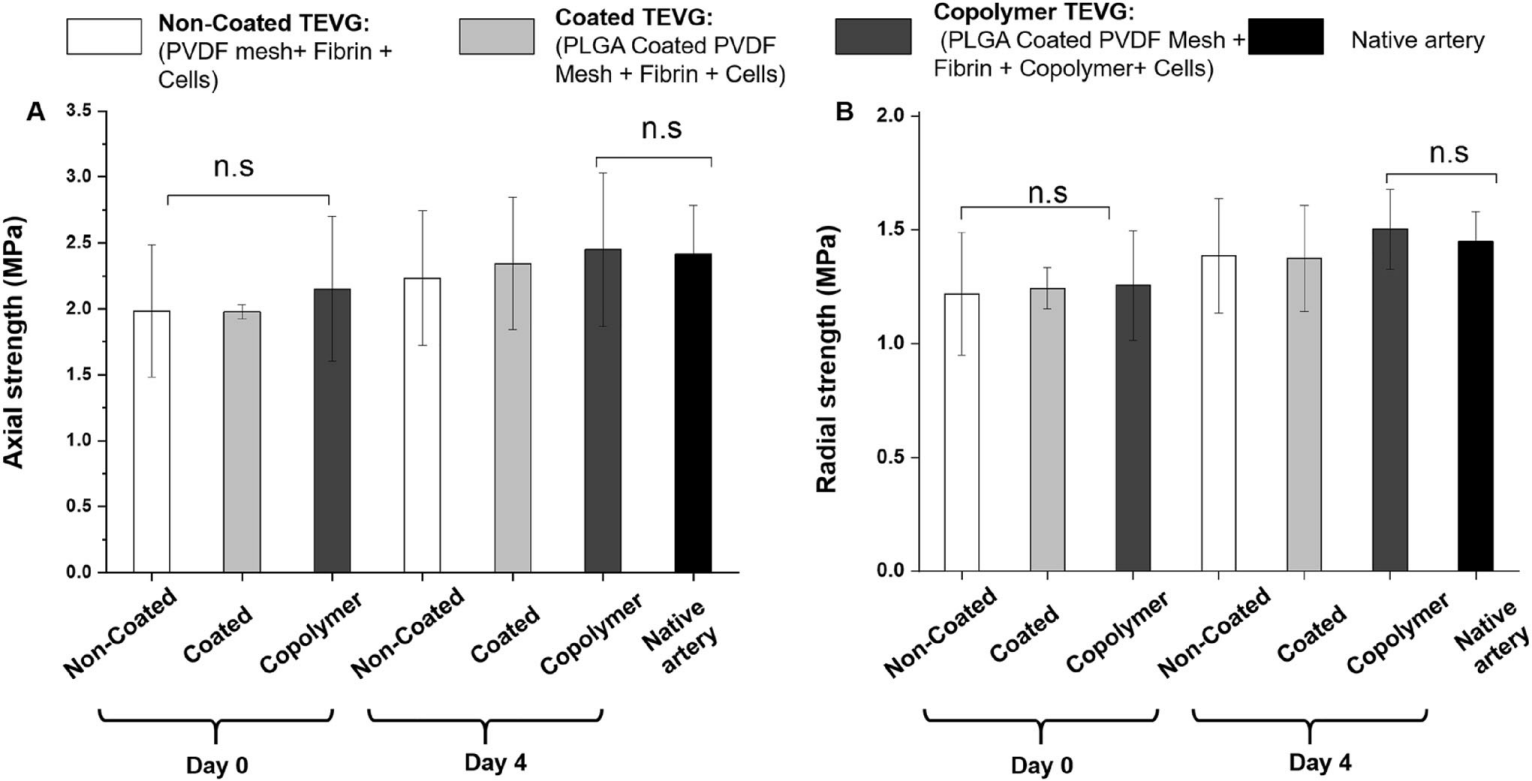
Biaxial test of the TEVGs. A) Axial strength, B) Radial strength. Statistical analysis was performed using one way ANOVA (mean ± SD) with Tukey post hoc corrections (n.s *p* > 0.05, *p ≤ 0.05,**p ≤ 0.01, ***p ≤ 0.001)

The axial strength of all TEVGs (non-coated, coated, and copolymer) was found to be higher than the radial strength. In comparison to the native artery, the TEVGs at day 0 show a lower strength, whereas the TEVGs at day 4 (n = 6) show a higher strength in both the axial and radial measurements. However, the difference is not statistically significant in both cases (Fig. 4A, B). Though the coating and copolymer addition in the TEVGs have no significant impact on enhancing the biaxial tensile strength, it indicates an increasing order. The highest tensile strength was found on the day 4 of copolymer-based TEVGs, where 2.45 ± 0.58 MPa axial strength and 1.50 ± 0.18 MPa radial strength were recorded.

The Youngs’s modulus of the native artery was found to be 1.5 ± 0.81 MPa which was the lowest in comparison to all sets of TEVGs (Fig. 8D). No significant differences were observed between the day 0 coted, non-coated, and copolymer samples. However, the highest young’s modulus, 12.31 ± 3.17 MPa, was recorded on the copolymer TEVG at day 4 samples.

### Immunohistochemistry

**Fig. 5:**
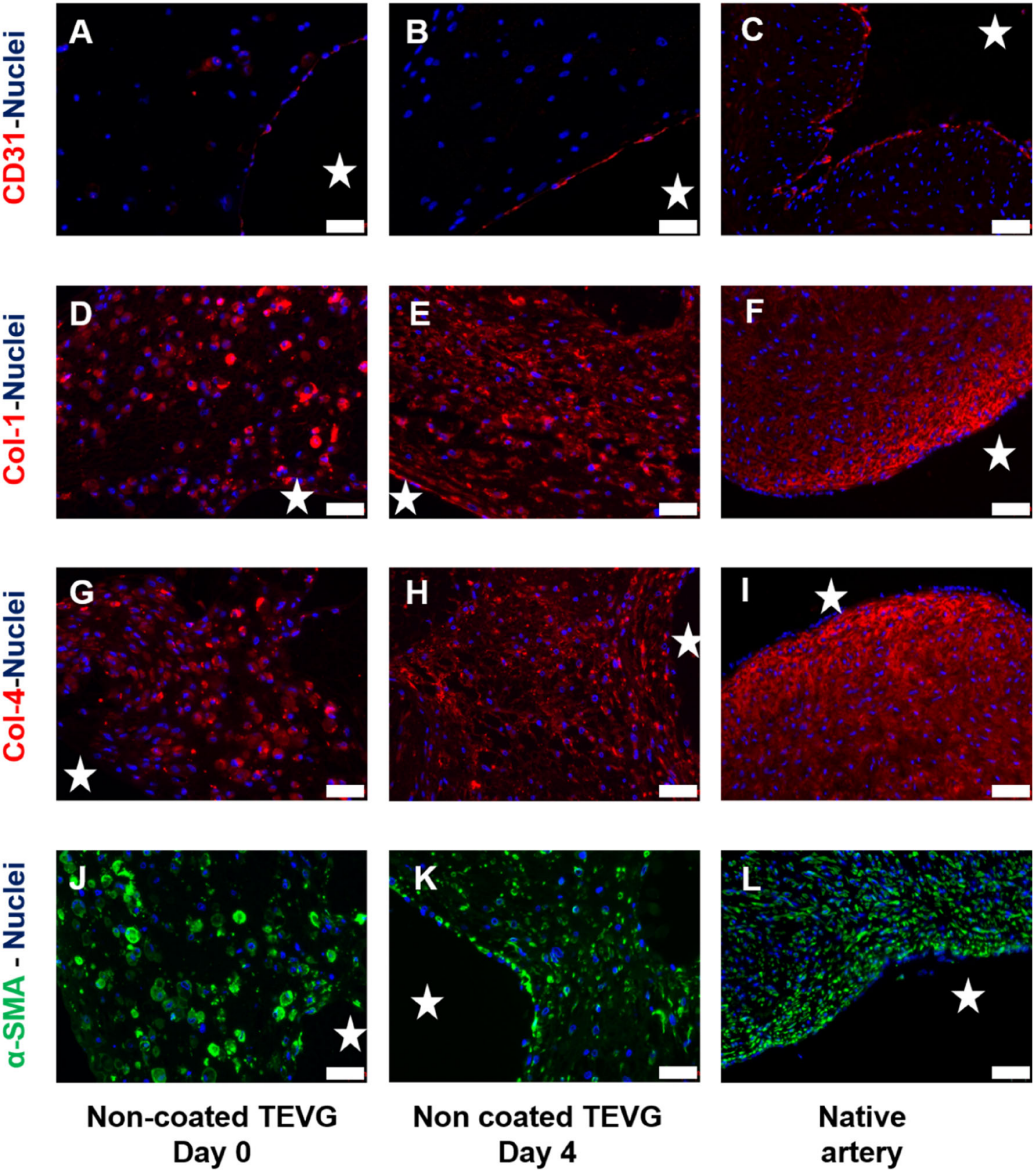
Immunohistochemical analysis of the non-coated TEVGs in comparison to a native artery (human umbilical cord). **A**–**C** CD31, DAPI. **D**–**F** Collagen 1, DAPI. **G**–**I** Collagen 4, DAPI. **J**–**L** Alpha SMA, DAPI. Scale bar 20 µm. The stars mark the lumen

**Fig. 6:**
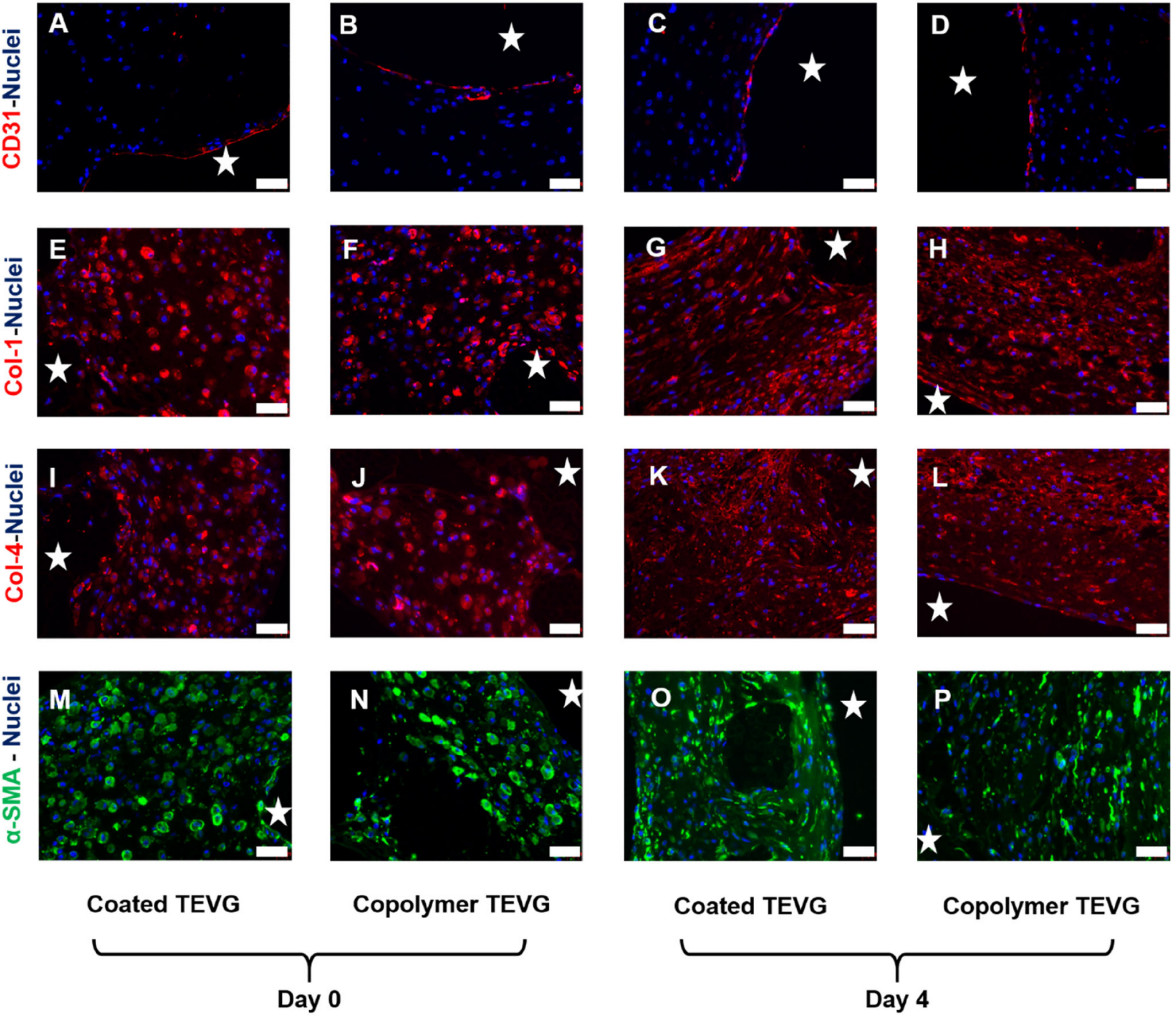
Immunohistochemical analysis of the coated and copolymer TEVGs. **A**–**D** CD31, DAPI, **E**–**H** Collagen 1, DAPI. **I**–**L** Collagen 4, DAPI. **M**–**P** Alpha SMA, DAPI. Scale bar 20 µm. The stars mark the lumen

TEVGs at day 0 showed low expression of collagen type I and type IV and smooth muscle actin. The bioreactor conditioning assisted in expressing the tissue maturation in the TEVGs at day 4. A monolayer of endothelial cells was found in all TEVGs, as evidenced by CD 31 staining. Although the signal of CD31 staining in TEVGs at day 0 was not very intense but TEVGs at day 4 had qualitative progress, and the endothelial layer could be observed clearly (Fig. 5). From the Alpha smooth muscle actin staining, sphere-shaped actin matrices were found near the smooth muscle cells in the freshly molded TEVGs, whereas in the matured TEVGs the geometrical shapes were found to be elliptical or flattened, which were similar to the native artery. Neither the coating nor the copolymer addition has any negative effect on the morphology or growth of the cells (Fig. 6).

**Fig. 7:**
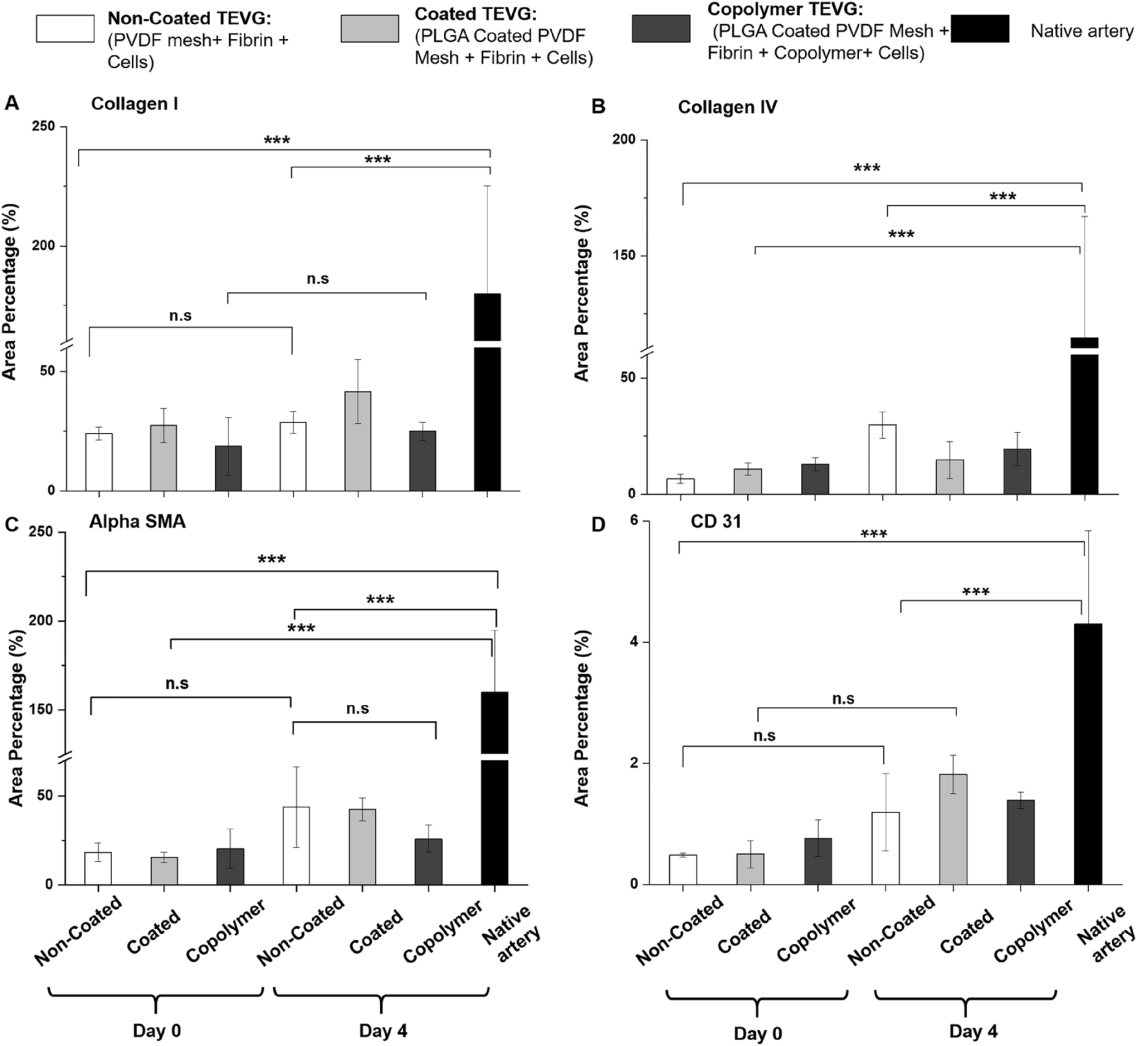
Quantitative analysis of the histology. **A** Collagen I, **B** collagen IV, **C** Alpha SMA D CD31. Statistical analysis was performed using one way ANOVA (mean ± SD) with Tukey post hoc corrections (n.s *p* > 0.05, *p ≤ 0.05,**p ≤ 0.01, ***p ≤ 0.001)

The quantitative analysis of immunohistology showed the native artery has the highest area percentage meaning more ECM across all the stainings (Fig. 7A–D). The TEVGs at day 0 have the lowest area percentage, and at day 4 increased area percentage in collagen I, collagen IV, alpha SMA, and CD 31 stainings, but the difference is statistically insignificant.

### ^1^H/^19^F Magnetic Resonance Imaging

**Fig. 8:**
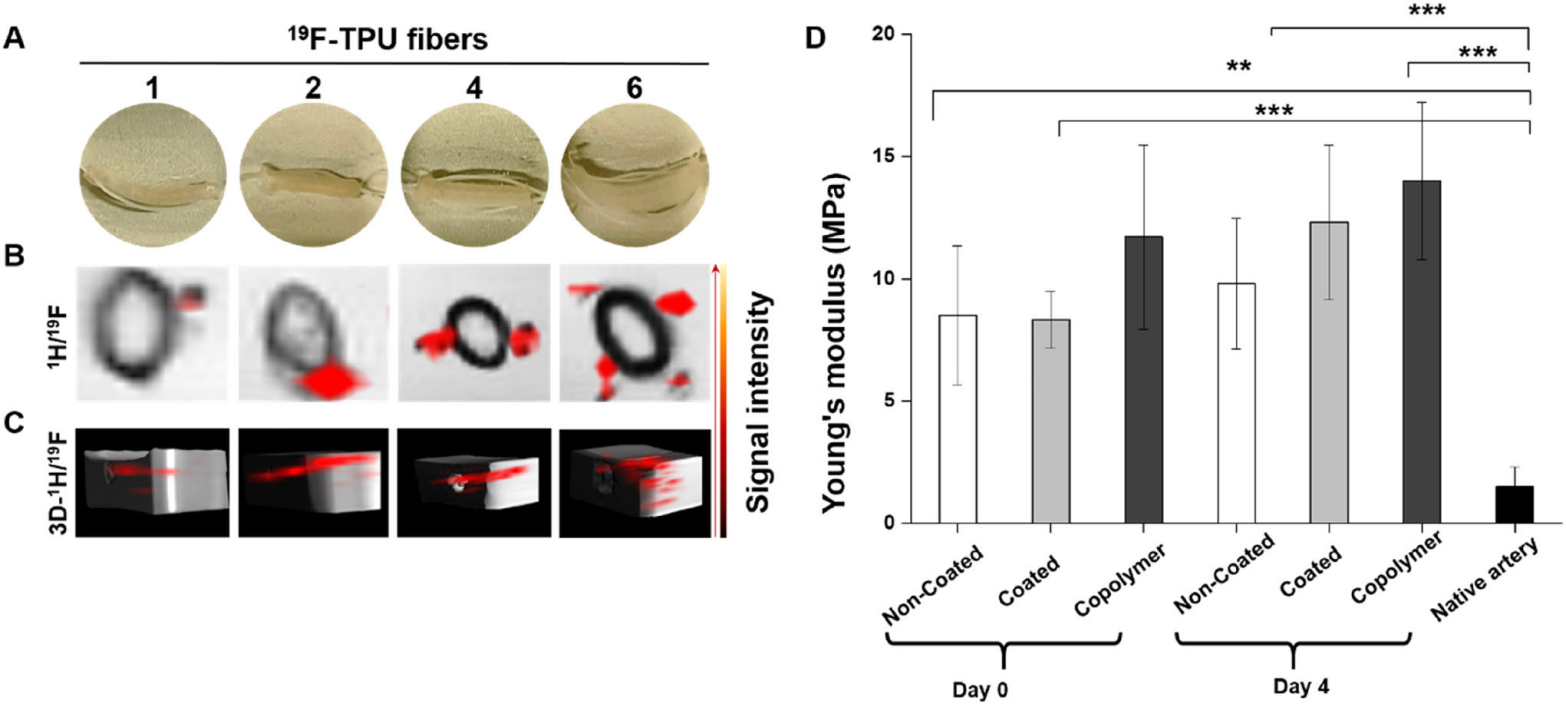
^19^F-TPU visibility via MRI. **A** Gelatin phantoms containing 0.2% (w/w) of SPION-labeled PLGA electrospun onto a PVDF warp-knitted scaffold combined with a different number of ^19^F-TPU fibers. **B** Transversal planes of superimposed grayscale T2W and “hot spot” UTE images show the increased ^19^F signal intensity correlating to a higher number of ^19^F-TPU fibers. **C** 3D rendering of superimposed ^1^H/^19^F-MR images of the samples. **D** Young’s modulus of the TEVGs and native artery. Statistical analysis was performed using one way ANOVA (mean ± SD) with Tukey post hoc corrections (n.s *p* > 0.05, *p ≤ 0.05, **p ≤ 0.01, ***p ≤ 0.001)

As illustrated in Fig. 8A, a systematic study was carried out to determine the ideal amount of ^19^F-TPU fibers needed to generate an adequate signal-to-noise ratio (SNR). Therefore, an increasing number of ^19^F-TPU fibers was combined with vascular graft textile scaffolds, composed of PVDF warp-knitted structure and 0.2% (w/w) SPION-labeled PLGA. The superimposition of qualitative T2W and UTE images showed both enhanced visibility of the PLGA layer due to the SPION labeling and increased ^19^F signal intensity corresponding to a higher number of ^19^F-TPU fibers (Fig. 8B). Interestingly, both signals are concomitantly detectable and, most importantly, distinguishable without interferences. As additionally shown through the 3D-rendering of the gelatin phantoms, the incorporation of a higher number of ^19^F-TPU fibers determined a higher SNR, hence a very detailed 3D reconstruction.

## Discussion

In the present study, we describe a further developed concept of our biohybrid textile-reinforced vascular graft to significantly shorten the production time and yet increase the mechanical strength and tissue maturation process. This is an important step toward clinical translation. To achieve this goal, we adopted two strategies which were successfully proved. The first hypothesis was that the initial burst strength could be increased by the application of an additional, electrospun layer of biodegradable PLGA fibers and the second was to integrate VP-*co*-GMA copolymer to mechanically strengthen the hydrogel as a cell carrier. Both strategies showed two-fold higher burst strength around 451 ± 81 mmHg than the non-coated TEVG 202 ± 24 mmHg when measured just after molding (day 0). After 4 days of bioreactor conditioning, the burst strength of copolymer TEVG was reached 617 ± 85 mmHg (Fig. 3E), and the coated TEVG to 586 ± 125 mmHg. Gui et al. and Syedain et al. have previously reported that the pulsatile flow while conditioning in a bioreactor helps in bringing higher bursts strength [18, 39]. Syedain et al. monitored the burst pressure of their vascular graft over time. After one week of cyclic flow, they burst at a pressure of about 270 mmHg and then increased to 596 ± 28 mmHg and 1366 ± 177 mmHg after 3 and 5 weeks respectively. Yao et al. has found burst pressure of 177 ± 5.3 mmHg on double-layered fibrin based TEVG which was not bioreactor conditioned and these TEVGs could be compared to the *day 0* TEVGs from our study [40]. Though the textile mesh in our study gives the backbone to support and higher mechanical strength but particularly the burst strength is more affected by the fibrin gel and the electrospun coating. Because the material failure point of a burst test specimen is similar to a pinhole (Fig. 3A, B), which is located in between the knitted fibers, the integration of PLGA coating and copolymer, therefore, increases the burst pressure. The pressure supplied underneath the sample to burst the sample could pass through the gel before it causes any damage to the textile. However, without the textile support, the fibrin gels are not strong enough and could lead to graft failure. Our production technique of biohybrid vascular graft has a design history, and we have already experimented with the long-term bioreactor conditioning. Having a similar construct, B. Tschoeke et al. has found 263 mmHg of burst pressure and 460 mmHg of burst pressure after 14 days and 21 days of bioreactor conditioning, respectively [22, 23].Wolf et al. have found 872 mmHg of burst pressure after 14 days of bioreactor conditioning by further modifying the bioreactor [24]. The burst strength increases when the TEVG is kept for a longer time. However, the main goal here is to initially enhance the burst strength before the complete ECM maturation by modifying the hydrogel with a copolymer and adding the extra electrospun layer. Doing more prolonged cultivation will shift our focus to achieving higher and higher native-like burst strength, which is not the immediate requirement in the clinic. The burst strength we gained (617 ± 85 mmHg) only in 4 days is six-fold higher than the systolic arterial pressure. The highest reported blood pressure on any human is 370 mmHg [41]. Our next focus will aim towards further reducing the bioreactor conditioning time and achieving peri-operative implantation.The perioperative tissue engineering method could become potentially the most vital approach to serve acute vascular dysfunction.

The suture retention strength of TEVGs was even higher than native artery because of the integrated textile reinforcement (Fig. 3F). The warp knitted textile mesh has a loop-like structure, which helps in retaining the suture for a longer time before failure. The bioreactor conditioning further positively influenced the suture retention strength as higher values were recorded on day 4 TEVG. The textile scaffold being the major factor here, its influence mainly determines the strength. Still, the combination of PLGA coating and copolymer in the TEVGs also has a slight impact, enhancing the suture retention strength. To obtain precise data for future clinical study, a 7.0 Prolene™ suture was used [42, 43]. Despite using small diameter sutures our vascular grafts have shown much higher suture retention strength than synthetic polymer-based vascular grafts [13, 44]. Even very high suture retention strength like 6.3 ± 2.8 N and 4.5 ± 1 N have been achieved by Tschoeke et al. and Dimopoulos et al., respectively, in different kinds of TEVGs [23, 45]. However, in these experiments, 4.0 prolene suture ware was used, whereas in vascular surgeries the use of 4.0 suture size has rarely been found in literature and the size is too big to be employed in small diameter vascular surgery. According to the DIN EN ISO 7198, the suture size must be close to clinical relevance to measuring the suture retention strength, hence a 7.0 size suture was used.

Though the tensile strength of vascular grafts has been widely studied by other researchers, biaxial studies are not found [46–48]. Some biaxial studies are done one after another axis but not pulled from both sides at the same time [49]. The axial strength of the TEVGs was higher than the radial strength because of the textile structure. The rhombus-like structure of the wrap knitting makes the axial pulling angle lesser than the radial, therefore resulting in higher tensile strength. The fibrin gel and the maturation have a lesser effect on the biaxial test because most of the strength develops from the textile mesh, unlike the burst strength study. However, when compared to the native artery, our TEVGs have similar tensile strength in both axial and radial directions. Young’s modulus was also significantly higher in TEVGs than in the native artery(1.5 ± 0.81 MPa). The nondegradable PVDF textile contributes more to the strength of the TEVGs, resulting in higher elastic modulus. Inoue et al. have recently discussed about the comparison of TEVGs with different native arteries. They tested and found 5.41 ± 1.16 MPa of Young’s modulus in raw TEVGs and after crosslinking by glutaraldehyde, they found 7.65 ± 1.18 MPa.Along with TEVGs, they have compared the young’s modulus of native veins and arteries and found 1.5 MPa and 3 MPa, respectively [50].

The production method of tissue-engineered grafts, the bioreactor, and the conditioning strategy plays a vital role in maintaining and growing the extracellular matrices [30, 51–53]. The immunohistological analysis has proved the development of essential extracellular matrices in just 4 days of bioreactor conditioning, thus, the molding technique and bioreactor used in the present study have a supportive and positive impact. The electrospun layer and copolymer have no negative impact on the ECM development, and on the other hand, they also do not show a further positive impact on ECM maturation, summarizing a neutral effect. Previous studies have shown that in the phase of tissue remodeling, the collagen amount could increase, decrease or remain the same depending on their enzyme activities [24, 52, 53]. In the present study, the collagen has shown a very good maturation in the TEVGs at day 4 than the TEVGs at day 0. The quantitative analysis of immunohistology shows a significant difference between native arteries and all sets of TEVGs across all the stainings. Achieving a similar ECM to the native artery in just 4 days was neither our hypothesis nor expected. Substantially shortening production time and yet having sufficient mechanical strength with increasing ECM was our expectation and the results obtained met our hypothesis.

In regards to the imaging approach, TEVG’s scaffold traceability was demonstrated by MRI. The PLGA degradation was monitored over 4 days and qualitatively analysed. The signal intensity was found to be weaker after 4 days of bioreactor conditioning, indirectly indicating the degradation of the PLGA coating. The monitoring of the degradation of this biocompatible TEVG component could be used as an indirect marker to predict patient compliance and ameliorate their outcomes [54, 55]. Subramanian et al. and Prabhakaran et al. had also previously discussed about the PLGA degradation in relation to tissue response [55, 56]. However, these studies were carried out via invasive imaging methods, thus after animal sacrifice or termination of the experiment. On the other hand, as shown in our previous publication, we recently proposed a novel imaging approach that showed the replacement of the degradable PLGA layer with new ECM deposition and the monitoring of the onset of inflammatory reaction in a non-invasive and non-destructive way [33]. Indeed, the longitudinal monitorability of the SPION-labeled PLGA textile component degradation was proved both *in vitro* and *in vivo* without harming or sacrificing the animals until the end of the experiment. Moreover, the ECM production and TEVG’s endothelial functionality were also investigated via non-invasive and non-destructive molecular MRI and targeted ultrasound. In addition, also here in our study, we showed the successful employment of this novel imaging approach to qualitatively assess the SPION-labeled PLGA layer degradation while TEVGs were still under bioreactor conditioning.

On the other hand, we were able to develop a novel textile scaffold by combining ^19^F-TPU and SPION-labeled PLGA, proving the visibility and detectability of both SPION-labeled PLGA and ^19^F-TPU fibers via ^1^H/^19^F MRI. During the last decades, several new strategies and approaches have been proposed to modify MRI scanners, customize coils and software, and develop fluorinated polymeric nanoparticles [57, 58] or metallic nanoparticles [59], micelles [60], nanoemulsions [61], or mesoporous silica spheres [62] as *in vivo* cell tracking and trafficking system [63] or to be integrated into tissue-engineered constructs [34, 64]. Therefore, the employment of non-invasive multimodal imaging modalities, as such we demonstrated herein to monitor the structure of our TEVGs, might provide tremendous advantages in several theranostic and therapeutic applications.

In conclusion, we here present a biohybrid tissue-engineered vascular graft with a distinct shortage of production time yet possessing sufficient mechanical strength. We were able to prove that the initial burst strength of the TEVGs can be enhanced by coating a layer of electrospun PLGA onto a PVDF textile mesh and integrating a VP-*co*-GMA copolymer into a fibrin cell carrier hydrogel. Applying this strategy allows to reduce the bioreactor conditioning time to 4 days instead of several weeks as just in 4 days of conditioning, sufficient ECM maturation can also be achieved.

Herein, we confirmed once again the increased visibility of our TEVGs after SPION-labeling and non-invasive qualitative assessment via 7T MRI. In addition, we demonstrated the employment of a novel imaging approach, which allowed the coupled visualization of SPION-labeled and ^19^F-TPU fibers via ^1^H/^19^F-MRI without interferences and overlapping of the signals.

In conclusion, the several strategies proposed throughout this study to effectively shorten TEVGs bioreactor conditioning time without jeopardizing their mechanical strength and to develop novel non-invasive and non-destructive multimodal imaging approaches to increase the visibility and monitorability of our vascular prostheses might strongly foster the *in vivo* applicability of our model of biohybrid tissue-engineered vascular grafts.

## Supplementary Information

Below is the link to the electronic supplementary material.

Supplementary file1 (DOCX 236 kb)
