# Supplementary material for: From In Vitro to Perioperative Vascular Tissue Engineering: Shortening Production Time by Traceable Textile-Reinforcement
Source: Tissue Eng Regen Med. 2022 Oct 6;19(6):1169–84. doi: 10.1007/s13770-022-00482-0 (PMC9679079; doi:10.1007/s13770-022-00482-0)
Supplement: Supplementary file 1 — Supplementary file1 (DOCX 236 kb) [file 13770_2022_482_MOESM1_ESM.docx]

**Supplementary information:**

***Table S1****:* p values obtained from the statistical analysis. Column 1 indicates the combinations, and the other columns (2-5) show the P value data for the performed tests*.*

| **combinations** | **P values** | | | |
| --- | --- | --- | --- | --- |
|  | Burst strength  test | Suture  retention  stregth | axial s  trength | radial strength |
| Coated TEVG day 0 , Not Coated TEVG day 0 | 1,36732 | 0.9479 | 1,00 | 0.99999 |
| CO-Polymer TEVG day 0, Not Coated TEVG day 0 | 0,0790723 | 0.51739 | 0.99587 | 0.99992 |
| CO-Polymer TEVG day 0 , Coated TEVG day 0 | 0.95667 | 0.97741 | 0.99495 | 1 |
| Not Coated TEVG day 4 , Not Coated TEVG day 0 | 98,6148 | 0.0255 | 0.96804 | 0.80529 |
| Not Coated TEVG day 4, Coated TEVG day 0 | 0.77632 | 0.23255 | 0.9639 | 0.89998 |
| Not Coated TEVG day 4 , CO-Polymer TEVG day 0 | 0.22348 | 0.71922 | 0.99993 | 0.93063 |
| Coated TEVG day 4, Not Coated TEVG day 0 | 0,00194105 | 0.00636 | 0.83686 | 0.84742 |
| Coated TEVG day 4 , Coated TEVG day 0 | 0.03517 | 0.08015 | 0.82564 | 0.9283 |
| Coated TEVG day 4 , CO-Polymer TEVG day 0 | 0.27243 | 0.39229 | 0.99128 | 0.95269 |
| Coated TEVG day 4, Not Coated TEVG day 4 | 69,3623 | 0.99821 | 0.99958 | 1 |
| CO-Polymer TEVG day 4, Not Coated TEVG day 0 | 0,00112218 | 0.00244 | 0.6135 | 0.24159 |
| CO-Polymer TEVG day 4 , Coated TEVG day 0 | 0.00483 | 0.03586 | 0.59867 | 0.34481 |
| CO-Polymer TEVG day 4 , CO-Polymer TEVG day 0 | 0.05848 | 0.22314 | 0.92517 | 0.39716 |
| CO-Polymer TEVG day 4, Not Coated TEVG day 4 | 0,73362 | 0.97421 | 0.9836 | 0.95434 |
| CO-Polymer TEVG day 4 , Coated TEVG day 4 | 0.9874 | 0.99985 | 0.99969 | 0.93276 |
| Native artery , Not Coated TEVG day 0 | 0 | 0.99964 | 0.68647 | 0.48046 |
| Native artery, Coated TEVG day 0 | 0 | 0.79278 | 0.67211 | 0.61618 |
| Native artery , CO-Polymer TEVG day 0 | 0 | 0.29106 | 0.95524 | 0.67546 |
| Native artery , Not Coated TEVG day 4 | 0 | 0.00905 | 0.99281 | 0.99809 |
| Native artery , Coated TEVG day 4 | 0 | 0.00209 | 0.99996 | 0.99564 |
| Native artery , CO-Polymer TEVG day 4 | 0 | 77,61 | 1,00 | 0.9993 |

Top view of a TEVG:

Figure S 1


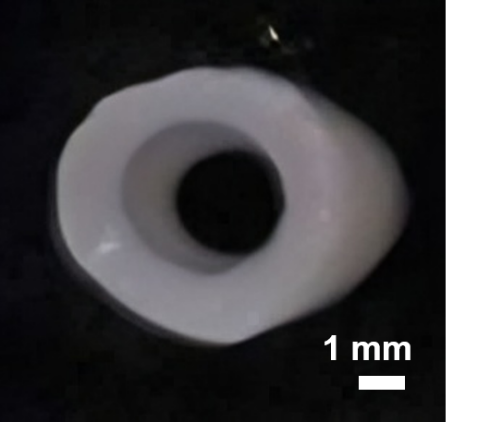


**Figure S1:**: Top view of a TEVG after molding
